# Pericardial Disease-Related Mortality in the United States: A Comprehensive Analysis of 1999 to 2019 Trends

**DOI:** 10.1016/j.jacadv.2025.102157

**Published:** 2025-09-15

**Authors:** Taimor Mohammed Khan, Muhammad Moiz Nasir, Saad Ahmed Waqas, Muhammad Salik Uddin, Aymen Ahmed, Muhammad Omar, Raheel Ahmed, Hasan Fareed Siddiqui

**Affiliations:** a Department of Medicine, Dow University of Health Sciences, Karachi, Pakistan; b Department of Medicine, Aga Khan University, Karachi, Pakistan; c National Heart and Lung Institute, Imperial College London, London, United Kingdom

**Keywords:** CDC WONDER, epidemiology, health disparities

## Abstract

**Background:**

Despite advancements in medical management, pericardial disease (PD)–related mortality is on the rise, yet comprehensive data on its prevalence and mortality rates in the United States remains limited.

**Objectives:**

This study aimed to analyze PD-related mortality trends in the United States from 1999 to 2019, focusing on demographic variations based on sex, age, race/ethnicity, geographical regions, and urban–rural distinctions.

**Methods:**

A descriptive analysis was conducted using death certificate data from the Centers for Disease Control and Prevention Wide-Ranging Online Data for Epidemiologic Research database. Mortality rates were assessed among adults aged ≥25 years diagnosed with PD, using International Classification of Diseases-10th Revision codes. Crude mortality rates and age-adjusted mortality rates (AAMRs) were calculated, and temporal trends were analyzed using joinpoint regression.

**Results:**

A total of 87,285 PD-related deaths were recorded during this period. AAMR initially decreased from 2.36 in 1999 to 1.70 in 2012, with an annual percentage change of −2.74% (*P* < 0.001) However, this trend reversed, as AAMR increased significantly to 2.04 by 2019, with an annual percentage change of +2.94% (*P* < 0.001). Higher mortality rates were observed among males, older adults (≥65 years), and non-Hispanic Black or African American individuals. The Western region exhibited the highest AAMR, whereas rural areas showed slightly elevated mortality rates compared to urban areas after 2012.

**Conclusions:**

These findings underscore a resurgence in PD-related mortality, highlighting the urgent need for improved public health strategies to address this growing burden, particularly among vulnerable populations.

Pericardial diseases (PDs) encompass a spectrum of conditions affecting the pericardium including acute pericarditis, constrictive pericarditis, pericardial effusion, hemopericardium, and cardiac tamponade. Among these, acute pericarditis is the most common initial presentation and often serves as the precursor to more severe complications if left untreated. Although this initial presentation itself is associated with significant morbidity, its progression to complications such hemopericardium and cardiac tamponade can lead to critical outcomes, including death.^1^

Pericarditis alone represents a significant health burden in the United States, accounting for approximately 5% of emergency department visits for nonischemic chest pain.^2^ Recurrent episodes occur in up to 30% of patients,^1^^,^^3^^,^^4^ with treatment often extending between 4.7 and 6.2 years.^5^ Beyond pericarditis, the broader spectrum of PD carry substantial clinical significance. Community-based studies have identified pericardial effusions in approximately 6% to 6.5% of adults, with prevalence rising to 15% among older individuals and up to 20% in hospitalized patients.^6^ Constrictive pericarditis, although rare, is associated with an in-hospital mortality of 7%, and if left untreated, may lead to mortality rates as high as 90%.^7^ Importantly, PD also frequently complicate systemic illnesses. Malignant pericardial effusion, seen in up to 21% of cancer patients, carry a 1-year mortality rate of 86%, with approximately 33% of patients dying within 30 days.^8^

Although prior research has characterized long-term trends and disparities in conditions, such as acute pericarditis^9^ and hemopericardium,^10^^,^^11^ comprehensive evaluations across the full spectrum of pericardial pathologies remain unexplored. By including all pericardial pathologies, our broader PD definition aims to capture mortality patterns that may have been overlooked in condition-specific studies. Given the potential severity of PD, understanding demographic and regional disparities is crucial. This study aims to describe differences in PD-related mortality among adults in the United States from 1999 to 2019, considering temporal trends and variations based on sex, age, race/ethnicity, geographical regions, and urban–rural distinctions.

## Methods

### Study setting and population

We conducted a descriptive analysis using death certificate data from the Centers for Disease Control and Prevention's (CDC) Wide-Ranging Online Data for Epidemiologic Research (WONDER) database.^12^ Our primary objective was to assess mortality rates among individuals aged ≥25 years diagnosed with PD from 1999 to 2019. To ensure an accurate evaluation of long-term trends, we excluded data post-2019 due to the widespread impact of the COVID-19 pandemic on overall mortality patterns. However, age-adjusted mortality rates (AAMRs) for this period were analyzed and reported separately to assess their distinct effects, as they may not reflect typical disease-specific trends. Affected individuals were identified using International Classification of Diseases, 10th Revision codes I30 and I31, which have been previously used to identify PD.^13^ Although the International Classification of Diseases-10th Revision code I32 (pericarditis in diseases classified elsewhere) is also conceptually relevant, it was not available in the CDC WONDER database and therefore, could not be included in the analysis. Our analysis focused on death certificates from the Multiple Cause of Death Public Use dataset, capturing cases where PD were listed as either an underlying or contributing cause of death. This approach was chosen to avoid underestimating mortality burden, as relying solely on the underlying cause may overlook the broader contribution of PD to death.^14^ Institutional Review Board approval was not required for this study, as we used a deidentified, publicly available data set. The Strengthening the Reporting of Observational Studies in Epidemiology guidelines for reporting observational studies were adhered to in this research.

### Data abstraction

We categorized data by demographic factors, including sex, age, race/ethnicity, urbanization level, state, census region, and place of death. Sex was defined as male or female. Age groups were defined as young adults (25-44 years), middle-aged adults (45-64 years), and older adults (65+ years), consistent with categories used in prior research with this database.^15^, ^16^, ^17^, ^18^ Racial/ethnic groups included Hispanic or Latinos, non-Hispanic (NH) Whites, NH Black or African Americans, and NH Asian or Pacific Islanders. Geographic classification followed the National Center for Health Statistics Urban-Rural Classification Scheme, categorizing areas as metropolitan or urban (population ≥50,000), and nonmetropolitan or rural (population <50,000). The United States was further divided into 4 regions based on the U.S. Census Bureau classification: Northeast, Midwest, South, and West. Locations of death were classified as medical facilities, decedent’s home, hospice facilities, nursing home/long-term care facilities, and other locations. There were no missing data for the variables of interest.

### Statistical analysis

We analyzed PD-related mortality trends from 1999 to 2019, stratified by year, sex, age, race, urbanization, and census region. Crude mortality rates (CMRs) and AAMRs per 100,000 individuals were computed. CMRs were determined by dividing the number of PD-related deaths by the corresponding U.S. population for each year, whereas AAMRs were computed by standardizing mortality rates to the 2000 U.S. population. AAMR was used for all analyses except those stratified by age categories, where CMR was used. Temporal trends were assessed separately for each stratum using the Joinpoint Regression Program (version 5.2.0, National Cancer Institute) by applying log-linear regression models to estimate the annual percent change (APC) and average APC (AAPC) in AAMR, with 95% CIs. The model determines the optimal number and location of joinpoints (ie, years where the slope of the mortality trend changes) resulting in one or more segments of time with distinct APCs. For most strata, the analysis identified 2 segments (1 joinpoint). The underlying model assumes Poisson variation in the rates; however, we applied the software’s built-in option for over dispersion correction to account for potential extra-Poisson variation. In addition, the model employed permutation tests with robust SEs to mitigate the influence of autocorrelation in the time series. These adjustments helped ensure reliable estimation of joinpoint and APC values. APCs were categorized as increasing or decreasing based on statistical deviation from the null hypothesis of zero change. Statistical significance was determined using a 2-tailed t-test with a significance threshold of *P* < 0.05.

## Results

A total of 87,285 PD-related deaths occurred between 1999 and 2019 among adults aged 25 years and older. Of these, the majority (69.2%) occurred in medical facilities, followed by decedents’ homes (21.0%), other locations (5.1%), nursing homes/long-term care facilities (3.1%), and hospice facilities (1.6%) (Supplemental Table 1).

### Annual trends for PD-related mortality

**Figure 1:**
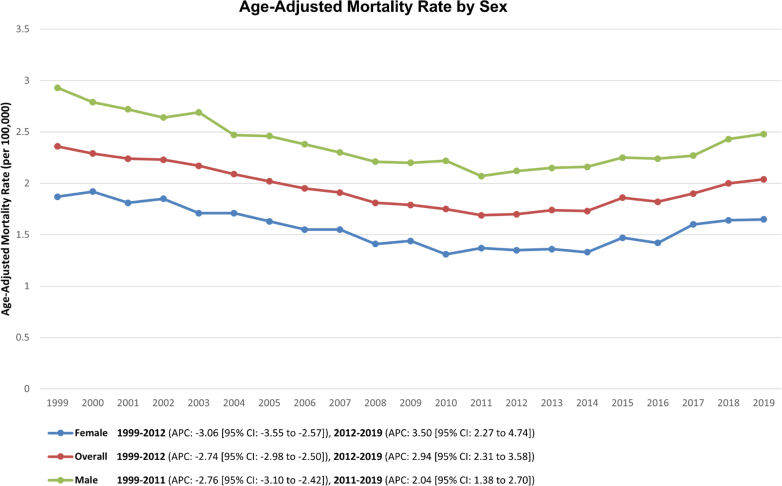
Overall and Sex-Wise Trends Trends in overall and sex-stratified PD-related mortality per 100,000 individuals in the United States from 1999 to 2019. APC = annual percentage change.

The AAMR for PD-related deaths in adults was 2.36 in 1999 and decreased to 1.70 by 2012 (APC: −2.74%; 95% CI: −3.02 to −2.50; *P* < 0.001). Following this, a significant increase in AAMR was seen, rising from 1.70 in 2012 to 2.04 by the end of the study period (APC: +2.94%; 95% CI: 2.31-3.81; *P* < 0.001) (Table 1, Figure 1, Supplemental Table 2).

**Table 1:** APC for Pericardial Disease-Related Mortality Among U.S. Adults Aged ≥25 years From 1999 to 2019

	Segment 1			Segment 2			Segment 3		
	Years	APC (95% CI)	*P* Value	Years	APC (95% CI)	*P* Value	Years	APC (95% CI)	*P* Value
Overall	1999-2012	−2.74 (−3.02 to −2.50)	<0.001	2012-2019	2.94 (2.31-3.81)	<0.001	-	-	-
Age group^a^									
25-44 y	1999-2011	−2.36 (−3.64 to −1.60)	<0.001	2011-2019	1.45 (0.33-3.59)	0.009	-	-	-
45-64 y	1999-2011	−2.10 (−3.02 to −1.47)	<0.001	2011-2019	2.06 (0.97-4.37)	<0.001	-	-	-
65+ y	1999-2012	−3.18 (−3.57 to −2.85)	<0.001	2012-2019	3.35 (2.55-4.46)	<0.001	-	-	-
Sex									
Female	1999-2012	−3.06 (−3.63 to −2.61)	<0.001	2012-2019	3.50 (2.38-4.92)	<0.001	-	-	-
Male	1999-2011	−2.76 (−3.12 to −2.44)	<0.001	2011-2019	2.04 (1.42-2.85)	<0.001	-	-	-
Race									
NH Asian/Pacific Islander	1999-2003	−9.23 (−22.85 to −1.29)	0.010	2003-2019	0.26 (−0.72 to 4.22)	0.383	-	-	-
NH Black/African American	1999-2012	−3.30 (−4.20 to −2.64)	<0.001	2012-2019	2.38 (0.77-5.15)	0.004	-	-	-
NH White	1999-2011	−2.48 (−4.51 to −1.21)	0.067	2011-2014	0.34 (−4.84 to 5.01)	0.867	2014-2019	3.77 (1.50-7.19)	0.028
Hispanic/Latino	1999-2012	−3.63 (−4.67 to −2.74)	<0.001	2012-2019	3.40 (2.24-6.67)	<0.001	-	-	-
Urbanization									
Metropolitan	1999-2012	−2.78 (−3.07 to −2.52)	<0.001	2012-2019	2.78 (2.17-3.70)	<0.001	-	-	-
Non-metropolitan	1999-2011	−2.49 (−3.21 to −1.92)	<0.001	2011-2019	3.30 (2.20-4.86)	<0.001	-	-	-
Census region									
Northeast	1999-2014	−2.37 (−2.83 to −1.90)	<0.001	2014-2019	3.70 (1.20-6.27)	<0.001	-	-	-
Midwest	1999-2012	−2.51 (−3.02 to −2.00)	<0.001	2012-2019	3.50 (2.15-4.88)	<0.001	-	-	-
South	1999-2011	−3.05 (−3.46 to −2.64)	<0.001	2011-2019	2.44 (1.72-3.17)	<0.001	-	-	-
West	1999-2011	−3.04 (−3.51 to −2.57)	<0.001	2011-2019	2.38 (1.55-3.21)	<0.001	-	-	-

Segments are derived from separate Joinpoint analyses for each demographic stratum. Most strata had 2 segments; NH White had 3. Blank cells indicate that the segment was not applicable because no additional Joinpoint was detected.

APC = annual percent change; NH = non-Hispanic.

a Crude mortality rates were used instead of AAMRs for age groups.

A similar trend was seen on sensitivity analysis, where PD were explicitly designated as the underlying cause of death, confirming a consistent decline in mortality rates followed by a late-period increase.

### PD-related AAMR stratified by demographics

#### Sex-wise analysis

During the study period from 1999 to 2019, female AAMR was consistently lower than male AAMR (overall AAMR for women: 1.57 [95% CI: 1.55-1.58]; overall AAMR for men: 2.37 [95% CI: 2.35-2.39]) (Supplemental Table 2).

A decreasing trend was noted for both men and women between 1999 and 2012, followed by a notable rise in AAMRs up to 2019. In 1999, the AAMR for women was 1.87, which decreased to 1.35 by 2012 (APC: −3.06%; 95% CI: −3.63 to −2.61; *P* < 0.001). This was followed by a rise to 1.65 by 2019 (APC: +3.50%; 95% CI: 2.38-4.92; *P* < 0.001). For men, AAMRs decreased from 2.93 in 1999 to 2.12 by 2012 (APC: −2.76%; 95% CI: −3.12 to −2.44; *P* < 0.001). This was followed by an increase in AAMR up to 2.48 by 2019 (APC: +2.04%; 95% CI: 1.41-2.85; *P* < 0.001) (Table 1, Figure 1).

#### Age group–wise analysis

From 1999 to 2019, the CMR per 100,000 for older adults (≥65 years) was notably higher than that for middle-aged adults (45-64 years), which was greater than that for younger adults (25-44 years). Specifically, the CMRs were 0.53 (95% CI: 0.52-0.54) for the younger group, 2.11 (95% CI: 2.09-2.13) for the middle-aged group, and 5.04 (95% CI: 4.99-5.08) for the older group (Supplemental Table 3).

All age groups exhibited declining trends from the start of the study till 2011 with the most prominent decrease seen in older adults (APC: −3.18%; 95% CI: −3.53 to −2.82; *P* < 0.001). After the initial period of decline, a rising trend in mortality rates was seen across all age groups with the most pronounced increase also seen in older adults (APC: +3.35%; 95% CI: 2.47-4.24; *P* < 0.001) (Table 1, Supplemental Figure 1).

#### Race-wise analysis

Throughout the study period from 1999 to 2019, NH Black or African American individuals consistently exhibited the highest overall AAMRs among all racial groups (2.68 [95% CI: 2.63-2.73]). They were followed by NH White individuals (1.89 [95% CI: 1.87-1.91]), Hispanic or Latino individuals (1.62 [95% CI: 1.58-1.66]) and NH Asian or Pacific Islanders (1.54 [95% CI: 1.50-1.58]) (Supplemental Table 4).

A decrease in AAMR was observed across all racial groups during the early period of the study. NH Asian or Pacific Islander individuals exhibited the steepest decline in mortality rates during this period (APC: −9.23%; 95% CI: −22.85 to −1.29; *P* = 0.010). They were followed by Hispanic or Latino individuals (APC: −3.63%; 95% CI: −4.67 to −2.74; *P* < 0.001), NH Black or African American individuals (APC: −3.30%; 95% CI: −4.20 to −2.64; *P* < 0.001), and NH White individuals (APC: −2.48%; 95% CI: −4.51 to −1.21; *P* = 0.067).

**Figure 2:**
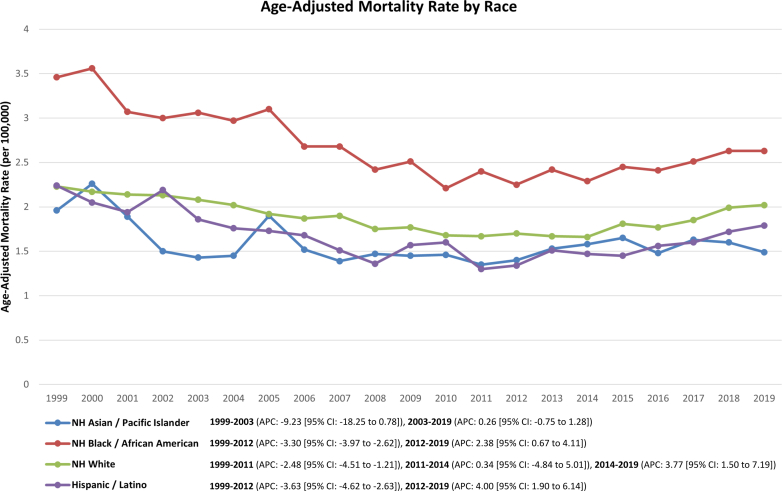
Race-Wise Trends Trends in PD-related mortality per 100,000 individuals, stratified by race in the United States from 1999 to 2019. NH = non-Hispanic; other abbreviations as in Figure 1.

After the period of decline, most racial groups experienced an increasing trend in AAMR until 2019. The most pronounced increase was seen in Hispanic or Latino individuals (APC: +3.40%; 95% CI: 2.24-6.67; *P* < 0.001) followed by NH White individuals ([first segment: APC: +0.34%; 95% CI: −4.84 to 5.01; *P* = 0.867] [second segment: APC: +3.77%; 95% CI: 1.51-7.19; *P* = 0.028]), followed by NH Black or African American individuals (APC: +2.38%; 95% CI: 0.77-5.15; *P* < 0.001). Contrastingly, NH Asian or Pacific Islander individuals saw a plateau in AAMR following the period of decline (APC: +0.26%; 95% CI: −0.72 to 4.22; *P* = 0.383) (Table 1, Figure 2).

### PD-related AAMR stratified by region

#### Urbanization-wise analysis

During the study period, both metropolitan and nonmetropolitan areas exhibited similar AAMRs related to PD (overall AAMR for metropolitan areas: 1.95 [95% CI: 1.93-1.96]; overall AAMR for nonmetropolitan areas: 1.98 [95% CI: 1.95-2.02]) (Supplemental Table 5).

**Figure 3:**
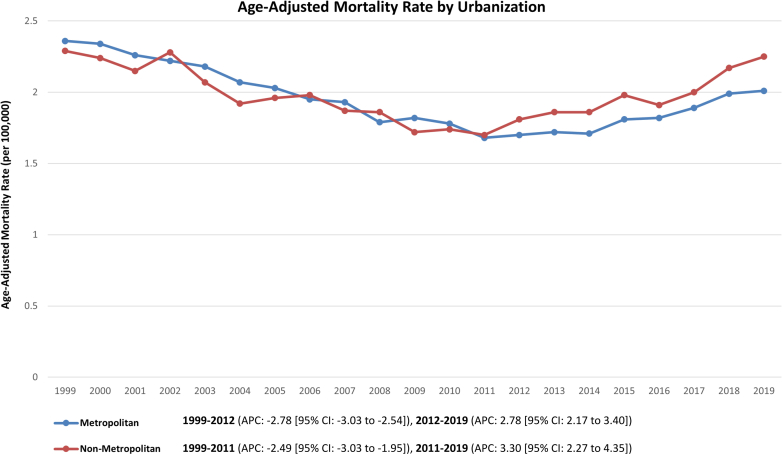
Urbanization-Wise Trends Trends in PD-related mortality per 100,000 individuals, stratified by the level of urbanization in the United States from 1999 to 2019. Abbreviations as in Figure 1.

A decline in AAMR was observed across both urbanization levels during the early study period, followed by an increasing trend till 2019. (AAPC for metropolitan areas: −0.87% [95% CI: −1.02 to −0.72; *P* < 0.001]; AAPC for nonmetropolitan areas: −0.21% [95% CI: −0.54 to 0.09; *P* = 0.162]) (Table 1, Figure 3).

#### State and census-wise analysis

**Figure 4:**
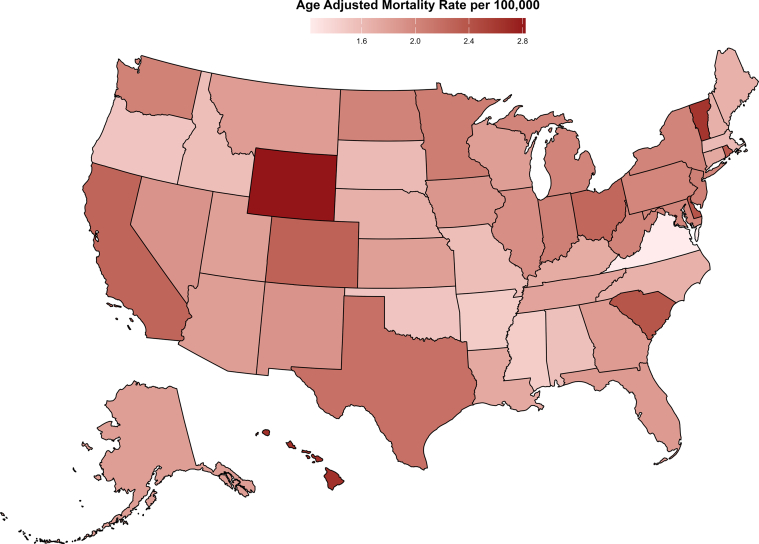
States Map PD-related mortality per 100,000 stratified by state in the United States from 1999 to 2019.

Similar AAMRs were observed among states, with values ranging from 1.22 in Virginia to 2.82 in Wyoming. States in the upper 90th percentile of PD-related deaths included Wyoming, District of Columbia, Hawaii, Vermont, and Delaware whereas states in the lower 10th percentile included Oregon, Arkansas, Mississippi, Alabama, and Virginia (Figure 4, Supplemental Table 6).

When comparing AAMRs across different geographic regions, the highest mortality was observed in the Western region, which had an AAMR of 2.11 (95% CI: 2.08-2.13) followed by the Northeast with an AAMR of 1.97 (95% CI: 1.94-2.00), the Midwest with an AAMR of 1.96 (95% CI: 1.93-1.99), and the South, with an AAMR of 1.82 (95% CI: 1.80-1.84) (Table 1, Supplemental Figure 2, Supplemental Table 7).

### PD as an underlying cause of death

Out of 87,285 PD-related deaths, 12,519 listed PD as the underlying cause of death. In the analysis limited to cases where PD were exclusively identified as the underlying cause, PD mortality decreased from 0.31 (95% CI: 0.28-0.34) in 1999, to 0.24 (95% CI: 0.22-0.26) in 2010 (APC: −1.32%; 95% CI: −5.31 to −0.30; *P* = 0.018) and then rose to 0.32 (95% CI: 0.30-0.35) by 2019 (APC: +2.28%; 95% CI: 0.40-9.42; *P* = 0.018) (Supplemental Figure 3).

Throughout the study period, men had a higher PD-related mortality rate compared to women (overall AAMR for men: 0.31 [95% CI: 0.31-0.32]; overall AAMR for women: 0.24 [95% CI: 0.23-0.25]) (Supplemental Table 8).

### PD-related mortality, during and post-COVID-19

As compared to the overall prepandemic AAMR of 1.94 (95% CI: 1.93-1.96), AAMRs rose to 2.28 (95% CI: 2.24-2.32) during the pandemic (2020-2021), and continued to increase postpandemic (2022-2023), rising to 2.48 (95% CI: 2.44-2.53).

## Discussion

The present study offers a 21-year analysis of death certificate data, for PD-related mortality among adults in the United States, sourced from the CDC WONDER database. Our study identifies a concerning rise in PD-related mortality following a period of decline from 1999 to 2012. Although AAMRs decreased steadily during this earlier period, the trend shifted after 2012, with mortality rates beginning to rise again.

In our analysis, males were found to experience higher mortality rates from PD than females, whereas NH Black or African American individuals had the highest mortality rates across all racial and ethnic groups. Furthermore, adults aged 65 and older exhibited the highest mortality rates among all age groups. Geographic analyses revealed a steady rise in mortality rates across all regions, following an initial period of decrease, with non-metropolitan areas showing slightly higher mortality rates compared to metropolitan areas (Central Illustration).

**Central Illustration:**
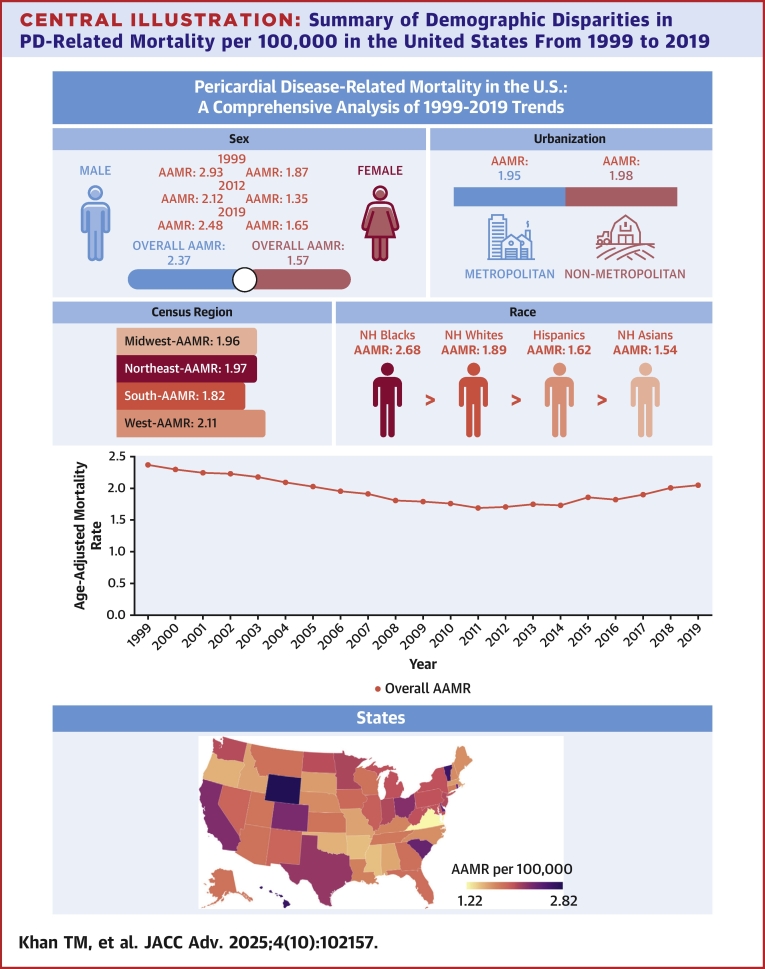
Summary of Demographic Disparities in PD-Related Mortality per 100,000 in the United States From 1999 to 2019 AAMR = age-adjusted mortality rate; NH = non-Hispanic.

The initial decrease in AAMRs from 1999 to 2012 can be explained by improvements in health care practices, including enhanced physician training in the diagnosis of PD, increased accessibility to echocardiograms, and an increased number of available cardiologists.^19^^,^^20^ However, the rise in PD-related mortality observed after 2012 has not been explained in previous literature. Several factors may have contributed, although these remain speculative and require further investigation. Notably, this increase was most pronounced among older adults, a group inherently at a higher risk for complications from PD.^21^ This trend may therefore be attributed, in part, to the aging U.S. population, as older adults now account for a growing segment of society. In addition, a surge in antiviral-resistant influenza virus specimens was documented during this period.^22^ Given that most idiopathic cases of acute pericarditis are viral in origin, this increase in resistant virus strains may have adversely affected patient outcomes.^23^ Moreover, a 7% rise in HIV infections from 2012 to 2016 may have also contributed to the increased mortality burden, as HIV-positive individuals are known to be at an elevated risk for PD.^23^^,^^24^ Although these observations may provide potential context for the reversal in mortality trends, further studies using individual-level clinical data are needed to substantiate these associations and clarify the underlying causes.

Although some progress has been made in understanding the mechanisms and risk factors for pericarditis, 80% to 90% of diagnoses, remain idiopathic.^1^ The pathophysiology involves inflammation of the pericardial sac, which can be triggered by infections (most commonly viral), autoimmune diseases, or postcardiac injury syndromes.^25^ In addition, comorbid conditions such as chronic kidney disease are well established as a significant risk factor for the development of pericarditis and subsequent complications.^26^

Throughout the study period, the AAMR for males consistently exceeded that for females, highlighting a persistent disparity in mortality rates between the sexes. Despite previous literature having identified this difference, what accounts for this disparity is still not well understood.^27^^,^^28^ Although our data set does not allow for causal inference, some factors have been proposed in the literature that may contribute. For instance, men experience a higher prevalence of chronic kidney disease which is known to complicate PD and increase mortality risk.^29^ In addition, men are more likely to present with severe forms of PD including pericardial effusion and tamponade,^30^ which can lead to worse outcomes. Hormonal differences have also been suggested, with testosterone potentially promoting a more intense inflammatory response that predisposes men to acute pericarditis.^31^^,^^32^ Furthermore, studies suggest that men are less likely to seek timely medical attention,^33^^,^^34^ potentially leading to delayed diagnosis and treatment, worsening outcomes. Although these explanations are plausible, they remain speculative and should be interpreted cautiously in the absence of individual-level data.

Among racial groups, NH Black or African Americans were observed to have the highest mortality rates; however other groups, including NH Whites and Hispanics or Latinos also experienced an increase in AAMR nearing the end of the study period. This disparity may be attributed to the fact African American individuals have a higher susceptibility for chronic kidney disease,^35^ due to which they have a higher incidence of uremic pericarditis compared to other populations.^36^ Furthermore, systemic factors—such as lower socioeconomic status, reduced access to high-quality care, and experiences of racial discrimination—may contribute to delays in diagnosis and treatment, potentially worsening outcomes.^36^

On urbanization-wise analysis, we observed that rural areas outpaced urban areas in mortality rates post-2012, which contrasts with the trend seen in the preceding years. Several inter-related factors may have contributed to this trend. Rural populations are known to carry a greater burden of underlying health conditions; for instance, nearly 24% of rural adults report significant health issues compared to just 3% in urban areas.^37^ This disparity in health status can lead to more severe complications from viral infections, including those that may precipitate pericarditis. In addition, rural populations tends to be older, with a higher percentage of individuals aged 65 and older, which may partly explain the observed increase in vulnerability.^37^ Limited health care access could also play a role, as many rural residents live more than 32 miles from an intensive care hospital, potentially delaying timely care for acute conditions such as pericarditis.^37^

Recently, 2 significant new etiologies of pericarditis have emerged: SARS-CoV-2 as an infectious cause and the COVID-19 mRNA vaccines as a noninfectious trigger.^38^^,^^39^ These associations have drawn increased attention to pericarditis, alongside myocarditis, in the context of the COVID-19 pandemic. Real-world data suggest that new-onset pericarditis occurred in 1.5% of COVID-19 cases, with a concerning 6-month all-cause mortality rate of 15.5% for patients with pericarditis compared to 6.7% for matched controls without the condition.^40^ These findings underscore the need for further investigation into the potential implications of COVID-19 and its vaccines on pericardial disease and emphasize the importance of awareness and timely management in such contexts.

Future research should focus on addressing the disparities in PD-related mortality, particularly among disproportionately affected populations such as NH Black or African American individuals and rural residents. Investigating the underlying mechanisms that contribute to the higher prevalence of chronic conditions, such as chronic kidney disease, in these populations is essential for developing targeted interventions. In addition, studies should explore the impact of social determinants of health, including access to health care, socioeconomic status, and experiences of discrimination, on PD outcomes. Understanding the role of viral infections, particularly in the context of recent findings linking acute pericarditis to COVID-19 vaccination and SARS-CoV-2 infection, is also crucial. This research could benefit public health strategies aimed at improving vaccination outreach and education, particularly in vulnerable communities. By addressing these multifaceted issues, future studies can contribute to more effective prevention and treatment strategies, ultimately improving health outcomes for those disproportionately affected by pericarditis.

### Study Limitations

The findings in this report are subject to certain limitations. First, this study relies on death certificate data from the CDC WONDER database, which may be subject to misclassification errors and lacks detailed clinical information such as comorbidities, treatment history, and disease severity. In addition, the absence of individual-level comorbidity data in the CDC WONDER database precludes direct assessment of the impact of conditions such as chronic kidney disease and HIV on PD-related mortality. Moreover, the study focuses exclusively on mortality data and does not capture the burden of recurrent or chronic pericarditis. Socioeconomic disparities and differences in health care access, which may influence outcomes, are also not accounted for. In addition, the study does not assess changes in treatment practices over time, and because the data set extends only through 2019, it does not include the potential effects of COVID-19-related pericarditis. Finally, the generalizability of these findings beyond the United States may be limited due to differences in population characteristics and health care systems internationally.

## Conclusions

PD-related mortality in the United States demonstrated a concerning shift over the past 2 decades, with an initial decline from 1999 to 2012 followed by a subsequent increase. This reversal highlights the urgent need to investigate factors contributing to this resurgence, particularly among high-risk populations such as older adults, males, NH Black or African American individuals, and rural residents.

Perspectives **COMPETENCY IN MEDICAL KNOWLEDGE:** Our study identified a significant reversal in PD-related mortality trends in the United States between 1999 and 2019. After years of decline, mortality rates began rising again in 2012. The burden is disproportionately higher among men, older adults, NH Black individuals, and those in rural or Western U.S. regions.

**TRANSLATIONAL OUTLOOK:** Targeted clinical and public health strategies are needed to address the rising burden of PD, with a focus on early detection, improved access to care, and equity-focused interventions in high-risk populations.

## Funding support and author disclosures

The authors have reported that they have no relationships relevant to the contents of this paper to disclose.
