# Supplementary material for: Pericardial Disease-Related Mortality in the United States: A Comprehensive Analysis of 1999 to 2019 Trends
Source: JACC Adv. 2025 Sep 15;4(10):102157. doi: 10.1016/j.jacadv.2025.102157 (PMC12464709; doi:10.1016/j.jacadv.2025.102157)

**Supplemental Table 1.** PD-related mortality stratified by place of death in the United States from 1999 to 2019

| **Place of Death** | **Deaths** | **% of Total Deaths** |
| --- | --- | --- |
| **Medical Facility - Inpatient** | 44393 | 50.86% |
| **Medical Facility - Outpatient or ER** | 14623 | 16.75% |
| **Medical Facility - Dead on Arrival** | 1318 | 1.51% |
| **Medical Facility - Status unknown** | 89 | 0.10% |
| **Decedent's home** | 18293 | 20.96% |
| **Hospice facility** | 1399 | 1.60% |
| **Nursing home/long term care** | 2731 | 3.13% |
| **Other** | 4176 | 4.78% |
| **Place of death unknown** | 263 | 0.30% |
| **Total** | 87285 | 100.00% |

**Supplemental Table 2.** Overall and sex-stratified PD-related AAMR per 100,000 in the United States from 1999 to 2019.

| **Age-Adjusted Mortality Rate (95% CI)** | | | |
| --- | --- | --- | --- |
| **Year** | **Men** | **Women** | **Overall** |
| **1999** | 2.93 (2.81–3.05) | 1.87 (1.79–1.95) | 2.36 (2.29–2.43) |
| **2000** | 2.79 (2.67–2.90) | 1.92 (1.83–2.00) | 2.29 (2.22–2.36) |
| **2001** | 2.72 (2.61–2.84) | 1.81 (1.73–1.90) | 2.24 (2.17–2.31) |
| **2002** | 2.64 (2.53–2.75) | 1.85 (1.77–1.94) | 2.23 (2.16–2.29) |
| **2003** | 2.69 (2.58–2.80) | 1.71 (1.63–1.79) | 2.17 (2.10–2.23) |
| **2004** | 2.47 (2.36–2.57) | 1.71 (1.63–1.79) | 2.09 (2.03–2.16) |
| **2005** | 2.46 (2.36–2.56) | 1.63 (1.55–1.70) | 2.02 (1.96–2.08) |
| **2006** | 2.38 (2.28–2.48) | 1.55 (1.47–1.62) | 1.95 (1.89–2.01) |
| **2007** | 2.30 (2.20–2.40) | 1.55 (1.48–1.62) | 1.91 (1.85–1.97) |
| **2008** | 2.21 (2.12–2.31) | 1.41 (1.34–1.48) | 1.81 (1.75–1.86) |
| **2009** | 2.20 (2.10–2.29) | 1.44 (1.37–1.51) | 1.79 (1.74–1.85) |
| **2010** | 2.22 (2.12–2.31) | 1.31 (1.25–1.38) | 1.75 (1.69–1.81) |
| **2011** | 2.07 (1.98–2.16) | 1.37 (1.31–1.44) | 1.69 (1.63–1.74) |
| **2012** | 2.12 (2.03–2.21) | 1.35 (1.28–1.41) | 1.70 (1.65–1.76) |
| **2013** | 2.15 (2.06–2.24) | 1.36 (1.29–1.42) | 1.74 (1.69–1.80) |
| **2014** | 2.16 (2.07–2.25) | 1.33 (1.27–1.40) | 1.73 (1.68–1.79) |
| **2015** | 2.25 (2.16–2.34) | 1.47 (1.40–1.54) | 1.86 (1.80–1.91) |
| **2016** | 2.24 (2.15–2.33) | 1.42 (1.35–1.49) | 1.82 (1.76–1.87) |
| **2017** | 2.27 (2.18–2.36) | 1.60 (1.53–1.67) | 1.90 (1.85–1.96) |
| **2018** | 2.43 (2.34–2.52) | 1.64 (1.57–1.71) | 2.00 (1.94–2.05) |
| **2019** | 2.48 (2.38–2.57) | 1.65 (1.58–1.72) | 2.04 (1.98–2.09) |
| **Total** | 2.37 (2.35–2.39) | 1.57 (1.55–1.58) | 1.94 (1.93–1.96) |

**Supplemental Table 3**. PD-related CMR stratified by age groups in the United States from 1999 to 2019

| **Crude Mortality Rate (95% CI)** | | | |
| --- | --- | --- | --- |
| **Year** | **Young Adults** | **Middle-Aged Adults** | **Old Adults** |
| **1999** | 0.54 (0.65-0.03) | 2.26 (2.51-0.06) | 6.20 (6.73-0.14) |
| **2000** | 0.53 (0.64-0.03) | 2.25 (2.50-0.06) | 5.96 (6.49-0.13) |
| **2001** | 0.56 (0.67-0.03) | 2.18 (2.42-0.06) | 5.65 (6.16-0.13) |
| **2002** | 0.55 (0.66-0.03) | 2.22 (2.45-0.06) | 5.58 (6.08-0.13) |
| **2003** | 0.52 (0.62-0.03) | 2.17 (2.39-0.06) | 5.47 (5.97-0.13) |
| **2004** | 0.51 (0.62-0.03) | 2.09 (2.31-0.06) | 5.13 (5.61-0.12) |
| **2005** | 0.49 (0.59-0.03) | 2.09 (2.30-0.05) | 5.08 (5.56-0.12) |
| **2006** | 0.49 (0.59-0.03) | 2.01 (2.21-0.05) | 4.83 (5.29-0.12) |
| **2007** | 0.49 (0.60-0.03) | 2.04 (2.25-0.05) | 4.59 (5.04-0.11) |
| **2008** | 0.44 (0.54-0.02) | 1.87 (2.06-0.05) | 4.40 (4.83-0.11) |
| **2009** | 0.45 (0.54-0.02) | 1.85 (2.05-0.05) | 4.43 (4.85-0.11) |
| **2010** | 0.43 (0.53-0.02) | 1.82 (2.01-0.05) | 4.32 (4.73-0.11) |
| **2011** | 0.41 (0.50-0.02) | 1.80 (1.98-0.05) | 4.18 (4.58-0.10) |
| **2012** | 0.43 (0.52-0.02) | 1.90 (2.09-0.05) | 4.09 (4.48-0.10) |
| **2013** | 0.46 (0.55-0.02) | 1.83 (2.02-0.05) | 4.21 (4.59-0.10) |
| **2014** | 0.41 (0.50-0.02) | 1.92 (2.12-0.05) | 4.18 (4.56-0.10) |
| **2015** | 0.45 (0.54-0.02) | 1.91 (2.10-0.05) | 4.60 (4.99-0.10) |
| **2016** | 0.43 (0.53-0.02) | 1.94 (2.14-0.05) | 4.39 (4.76-0.10) |
| **2017** | 0.47 (0.57-0.02) | 1.99 (2.19-0.05) | 4.70 (5.09-0.10) |
| **2018** | 0.46 (0.56-0.02) | 2.12 (2.32-0.05) | 5.07 (5.46-0.10) |
| **2019** | 0.49 (0.59-0.02) | 2.18 (2.39-0.05) | 5.07 (5.45-0.10) |
| **Total** | 0.52 (0.54-0.01) | 2.09 (2.13-0.01) | 4.99 (5.08-0.02) |

**Supplemental Table 4.** PD-related AAMR per 100,000 stratified by Race in the United States from 1999 to 2019.

| **Age-Adjusted Mortality Rate (95% CI)** | | | | |
| --- | --- | --- | --- | --- |
| **Year** | **NH Asian or Pacific Islander** | **NH Black or African American** | **NH White** | **Hispanic or Latino** |
| **1999** | 1.96 (1.55–2.37) | 3.46 (3.17–3.74) | 2.23 (2.15–2.31) | 2.24 (1.94–2.54) |
| **2000** | 2.26 (1.83–2.70) | 3.56 (3.28–3.85) | 2.17 (2.09–2.25) | 2.05 (1.77–2.33) |
| **2001** | 1.89 (1.51–2.28) | 3.07 (2.81–3.33) | 2.14 (2.07–2.22) | 1.94 (1.68–2.20) |
| **2002** | 1.50 (1.21–1.85) | 3.00 (2.74–3.25) | 2.13 (2.06–2.21) | 2.19 (1.92–2.47) |
| **2003** | 1.43 (1.14–1.78) | 3.06 (2.81–3.31) | 2.08 (2.01–2.16) | 1.86 (1.62–2.11) |
| **2004** | 1.45 (1.15–1.79) | 2.97 (2.72–3.21) | 2.02 (1.95–2.09) | 1.76 (1.53–1.99) |
| **2005** | 1.90 (1.56–2.23) | 3.10 (2.85–3.35) | 1.92 (1.85–1.99) | 1.73 (1.51–1.95) |
| **2006** | 1.52 (1.23–1.82) | 2.68 (2.45–2.90) | 1.87 (1.81–1.94) | 1.68 (1.48–1.89) |
| **2007** | 1.39 (1.12–1.66) | 2.68 (2.45–2.90) | 1.90 (1.83–1.96) | 1.51 (1.31–1.70) |
| **2008** | 1.47 (1.20–1.74) | 2.42 (2.20–2.63) | 1.75 (1.69–1.82) | 1.36 (1.18–1.54) |
| **2009** | 1.45 (1.18–1.72) | 2.51 (2.30–2.73) | 1.77 (1.70–1.83) | 1.57 (1.37–1.76) |
| **2010** | 1.46 (1.20–1.72) | 2.21 (2.01–2.41) | 1.68 (1.61–1.74) | 1.60 (1.42–1.79) |
| **2011** | 1.35 (1.11–1.59) | 2.40 (2.19–2.60) | 1.67 (1.60–1.73) | 1.30 (1.14–1.47) |
| **2012** | 1.40 (1.16–1.64) | 2.25 (2.06–2.45) | 1.70 (1.64–1.77) | 1.34 (1.18–1.50) |
| **2013** | 1.53 (1.29–1.77) | 2.42 (2.22–2.62) | 1.67 (1.61–1.74) | 1.51 (1.35–1.67) |
| **2014** | 1.58 (1.34–1.82) | 2.29 (2.10–2.48) | 1.66 (1.59–1.72) | 1.47 (1.31–1.63) |
| **2015** | 1.65 (1.41–1.90) | 2.45 (2.25–2.65) | 1.81 (1.75–1.88) | 1.45 (1.29–1.61) |
| **2016** | 1.48 (1.26–1.70) | 2.41 (2.21–2.60) | 1.77 (1.71–1.83) | 1.56 (1.40–1.72) |
| **2017** | 1.63 (1.41–1.85) | 2.51 (2.31–2.71) | 1.85 (1.79–1.91) | 1.60 (1.44–1.76) |
| **2018** | 1.60 (1.38–1.82) | 2.63 (2.43–2.83) | 1.99 (1.92–2.06) | 1.72 (1.56–1.88) |
| **2019** | 1.49 (1.28–1.69) | 2.63 (2.43–2.83) | 2.02 (1.95–2.09) | 1.79 (1.63–1.95) |
| **Total** | 1.54 (1.50–1.58) | 2.68 (2.63–2.73) | 1.89 (1.87–1.91) | 1.62 (1.58–1.66) |

**Supplemental Table 5.** PD-related AAMR per 100,000 stratified by Urban-Rural classification in the United States from 1999 to 2019.

| **Age-Adjusted Rate (95% CI)** | | |
| --- | --- | --- |
| **Year** | **Metropolitan** | **Nonmetropolitan** |
| **1999** | 2.36 (2.28-2.44) | 2.29 (2.12-2.45) |
| **2000** | 2.34 (2.26-2.42) | 2.24 (2.08-2.41) |
| **2001** | 2.26 (2.18-2.33) | 2.15 (1.99-2.31) |
| **2002** | 2.22 (2.15-2.30) | 2.28 (2.11-2.45) |
| **2003** | 2.18 (2.10-2.25) | 2.07 (1.91-2.22) |
| **2004** | 2.07 (2.00-2.14) | 1.92 (1.77-2.07) |
| **2005** | 2.03 (1.97-2.10) | 1.96 (1.81-2.11) |
| **2006** | 1.95 (1.88-2.01) | 1.98 (1.83-2.13) |
| **2007** | 1.93 (1.87-2.00) | 1.87 (1.73-2.02) |
| **2008** | 1.79 (1.73-1.85) | 1.86 (1.71-2.00) |
| **2009** | 1.82 (1.75-1.88) | 1.72 (1.59-1.86) |
| **2010** | 1.78 (1.71-1.84) | 1.74 (1.60-1.88) |
| **2011** | 1.68 (1.62-1.73) | 1.70 (1.57-1.84) |
| **2012** | 1.70 (1.64-1.76) | 1.81 (1.67-1.95) |
| **2013** | 1.72 (1.66-1.78) | 1.86 (1.71-2.00) |
| **2014** | 1.71 (1.65-1.76) | 1.86 (1.72-2.00) |
| **2015** | 1.81 (1.75-1.87) | 1.98 (1.83-2.12) |
| **2016** | 1.82 (1.76-1.88) | 1.91 (1.77-2.06) |
| **2017** | 1.89 (1.83-1.95) | 2.00 (1.85-2.15) |
| **2018** | 1.99 (1.93-2.05) | 2.17 (2.02-2.32) |
| **2019** | 2.01 (1.95-2.07) | 2.25 (2.09-2.40) |
| **Total** | 1.95 (1.93-1.96) | 1.98 (1.95-2.02) |

**Supplemental Table 6.** PD-related AAMR per 100,000 stratified by State in the United States from 1999 to 2019.

| **State** | **Age-Adjusted Rate (95% CI)** |
| --- | --- |
| **Alabama** | 1.55 (1.45-1.65) |
| **Alaska** | 1.82 (1.48-2.16) |
| **Arizona** | 1.82 (1.72-1.92) |
| **Arkansas** | 1.46 (1.34-1.58) |
| **California** | 2.27 (2.23-2.31) |
| **Colorado** | 2.29 (2.17-2.41) |
| **Connecticut** | 1.74 (1.62-1.86) |
| **Delaware** | 2.40 (2.14-2.66) |
| **District of Columbia** | 2.78 (2.40-3.16) |
| **Florida** | 1.85 (1.81-1.89) |
| **Georgia** | 1.84 (1.76-1.92) |
| **Hawaii** | 2.67 (2.45-2.89) |
| **Idaho** | 1.57 (1.39-1.75) |
| **Illinois** | 1.94 (1.88-2.00) |
| **Indiana** | 2.06 (1.96-2.16) |
| **Iowa** | 1.88 (1.76-2.00) |
| **Kansas** | 1.81 (1.67-1.95) |
| **Kentucky** | 1.71 (1.61-1.81) |
| **Louisiana** | 1.73 (1.63-1.83) |
| **Maine** | 1.66 (1.48-1.84) |
| **Maryland** | 2.01 (1.91-2.11) |
| **Massachusetts** | 1.60 (1.52-1.68) |
| **Michigan** | 2.02 (1.94-2.10) |
| **Minnesota** | 2.08 (1.98-2.18) |
| **Mississippi** | 1.45 (1.33-1.57) |
| **Missouri** | 1.57 (1.49-1.65) |
| **Montana** | 1.83 (1.61-2.05) |
| **Nebraska** | 1.67 (1.51-1.83) |
| **Nevada** | 1.90 (1.76-2.04) |
| **New Hampshire** | 1.67 (1.49-1.85) |
| **New Jersey** | 2.06 (1.98-2.14) |
| **New Mexico** | 1.90 (1.74-2.06) |
| **New York** | 2.02 (1.96-2.08) |
| **North Carolina** | 1.66 (1.58-1.74) |
| **North Dakota** | 2.03 (1.75-2.31) |
| **Ohio** | 2.25 (2.17-2.33) |
| **Oklahoma** | 1.51 (1.41-1.61) |
| **Oregon** | 1.51 (1.41-1.61) |
| **Pennsylvania** | 2.01 (1.95-2.07) |
| **Rhode Island** | 2.37 (2.13-2.61) |
| **South Carolina** | 2.39 (2.27-2.51) |
| **South Dakota** | 1.60 (1.38-1.82) |
| **Tennessee** | 1.78 (1.70-1.86) |
| **Texas** | 2.18 (2.12-2.24) |
| **Utah** | 1.80 (1.64-1.96) |
| **Vermont** | 2.63 (2.31-2.95) |
| **Virginia** | 1.22 (1.16-1.28) |
| **Washington** | 2.04 (1.94-2.14) |
| **West Virginia** | 2.02 (1.86-2.18) |
| **Wisconsin** | 1.82 (1.72-1.92) |
| **Wyoming** | 2.82 (2.44-3.20) |

**Supplemental Table 7.** PD-related AAMR per 100,000 stratified by Census Region in the United States from 1999 to 2019.

| **Age Adjusted Mortality Rate (95% CI)** | | | | |
| --- | --- | --- | --- | --- |
| **Year** | **Northeast** | **Midwest** | **South** | **West** |
| **1999** | 2.38 (2.22-2.54) | 2.29 (2.15-2.44) | 2.23 (2.11-2.34) | 2.60 (2.43-2.76) |
| **2000** | 2.30 (2.14-2.45) | 2.30 (2.15-2.44) | 2.22 (2.10-2.34) | 2.54 (2.38-2.71) |
| **2001** | 2.39 (2.23-2.55) | 2.21 (2.07-2.36) | 2.08 (1.97-2.19) | 2.41 (2.26-2.57) |
| **2002** | 2.24 (2.09-2.39) | 2.21 (2.07-2.35) | 2.06 (1.95-2.17) | 2.47 (2.31-2.63) |
| **2003** | 2.19 (2.04-2.34) | 2.21 (2.07-2.35) | 1.98 (1.87-2.08) | 2.40 (2.25-2.55) |
| **2004** | 1.95 (1.81-2.09) | 2.11 (1.98-2.25) | 1.96 (1.86-2.07) | 2.20 (2.06-2.34) |
| **2005** | 2.08 (1.94-2.23) | 2.00 (1.87-2.13) | 1.93 (1.83-2.03) | 2.21 (2.07-2.35) |
| **2006** | 1.92 (1.78-2.06) | 1.85 (1.73-1.98) | 1.90 (1.80-2.00) | 2.14 (2.00-2.28) |
| **2007** | 2.01 (1.87-2.15) | 1.94 (1.81-2.07) | 1.75 (1.65-1.85) | 2.08 (1.94-2.21) |
| **2008** | 1.80 (1.67-1.93) | 1.88 (1.75-2.01) | 1.68 (1.58-1.77) | 1.87 (1.74-2.00) |
| **2009** | 1.87 (1.74-2.01) | 1.77 (1.65-1.89) | 1.71 (1.61-1.80) | 1.88 (1.75-2.00) |
| **2010** | 1.82 (1.69-1.95) | 1.72 (1.60-1.84) | 1.68 (1.59-1.77) | 1.88 (1.76-2.01) |
| **2011** | 1.78 (1.65-1.91) | 1.85 (1.73-1.97) | 1.50 (1.42-1.59) | 1.83 (1.71-1.95) |
| **2012** | 1.74 (1.62-1.87) | 1.64 (1.53-1.75) | 1.61 (1.53-1.70) | 1.95 (1.83-2.07) |
| **2013** | 1.71 (1.59-1.84) | 1.76 (1.64-1.88) | 1.65 (1.56-1.74) | 1.89 (1.77-2.01) |
| **2014** | 1.69 (1.56-1.81) | 1.81 (1.69-1.93) | 1.61 (1.52-1.69) | 1.90 (1.79-2.02) |
| **2015** | 1.80 (1.67-1.92) | 1.85 (1.73-1.97) | 1.76 (1.67-1.85) | 1.99 (1.87-2.11) |
| **2016** | 1.76 (1.64-1.89) | 1.85 (1.73-1.97) | 1.71 (1.62-1.80) | 1.99 (1.87-2.11) |
| **2017** | 1.73 (1.60-1.85) | 2.05 (1.93-2.18) | 1.83 (1.74-1.92) | 2.05 (1.93-2.17) |
| **2018** | 1.96 (1.83-2.09) | 2.16 (2.03-2.29) | 1.86 (1.77-1.95) | 2.18 (2.06-2.31) |
| **2019** | 2.02 (1.89-2.15) | 2.10 (1.98-2.23) | 1.93 (1.84-2.02) | 2.21 (2.08-2.33) |
| **Total** | 1.97 (1.94-2.00) | 1.96 (1.93-1.99) | 1.82 (1.80-1.84) | 2.11 (2.08-2.13) |

**Supplemental Table 8.** PD-related AAMR per 100,000 stratified by PD being the underlying cause of death in the United States from 1999 to 2019.

| **Year** | **Overall AAMR** | **Male AAMR** | **Female AAMR** |
| --- | --- | --- | --- |
| **1999** | 0.31 | 0.34 | 0.27 |
| **2000** | 0.26 | 0.32 | 0.21 |
| **2001** | 0.29 | 0.29 | 0.24 |
| **2002** | 0.32 | 0.35 | 0.25 |
| **2003** | 0.29 | 0.33 | 0.25 |
| **2004** | 0.28 | 0.32 | 0.26 |
| **2005** | 0.29 | 0.33 | 0.24 |
| **2006** | 0.25 | 0.33 | 0.23 |
| **2007** | 0.27 | 0.28 | 0.25 |
| **2008** | 0.27 | 0.3 | 0.22 |
| **2009** | 0.28 | 0.3 | 0.24 |
| **2010** | 0.24 | 0.29 | 0.2 |
| **2011** | 0.26 | 0.29 | 0.21 |
| **2012** | 0.26 | 0.26 | 0.2 |
| **2013** | 0.27 | 0.31 | 0.24 |
| **2014** | 0.29 | 0.32 | 0.22 |
| **2015** | 0.27 | 0.33 | 0.25 |
| **2016** | 0.3 | 0.33 | 0.22 |
| **2017** | 0.28 | 0.32 | 0.26 |
| **2018** | 0.29 | 0.34 | 0.27 |
| **2019** | 0.32 | 0.35 | 0.26 |
| **Total** | 0.28 | 0.31 | 0.24 |

**Supplemental Figure 1.** Age-adjusted mortality rate per 100,000 stratified by PD as the underlying cause of death in the United States from 1999 to 2019.


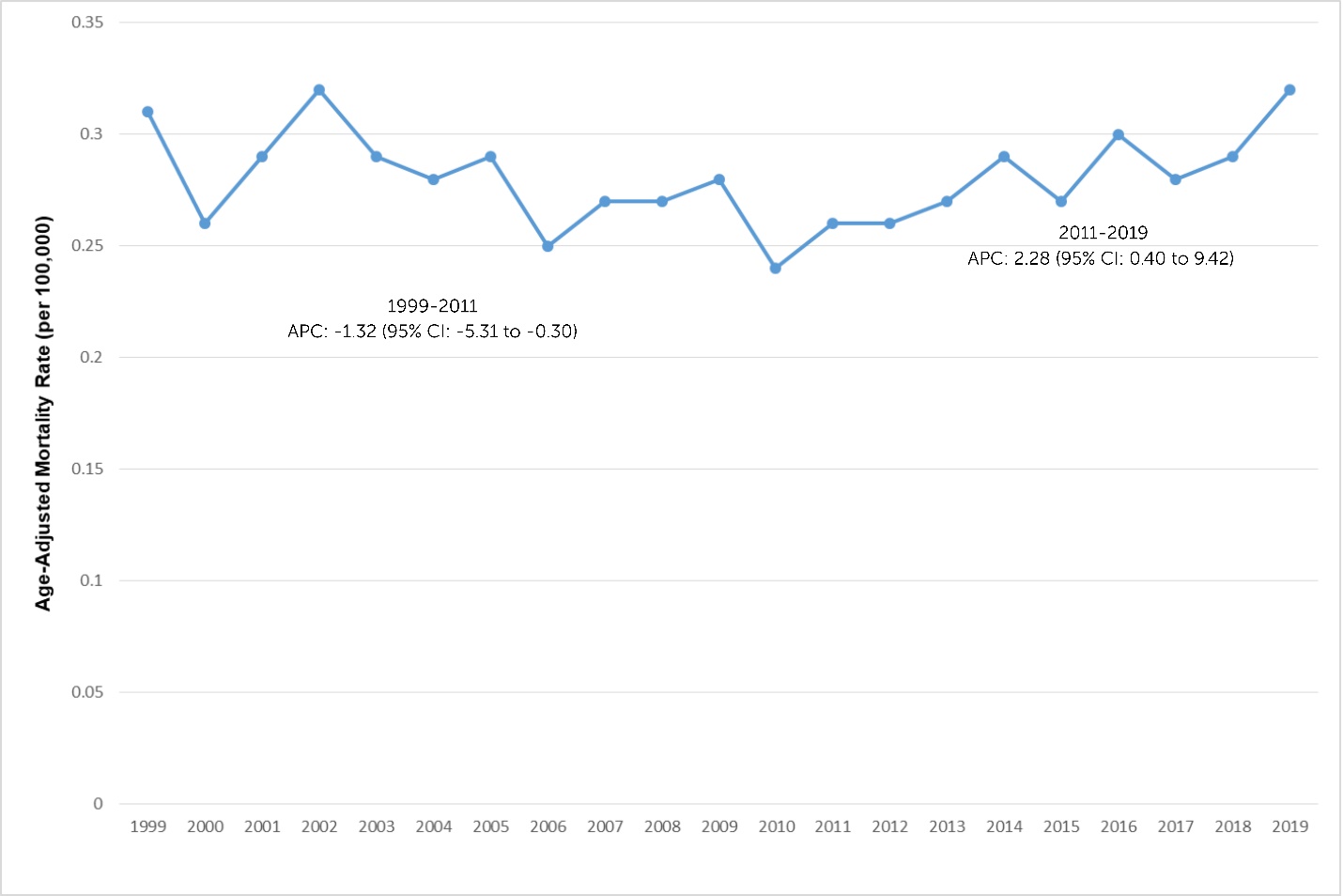


**Supplemental Figure 2.** Age-specific trends in PD-related mortality among adults in the United States from 1999 to 2019.


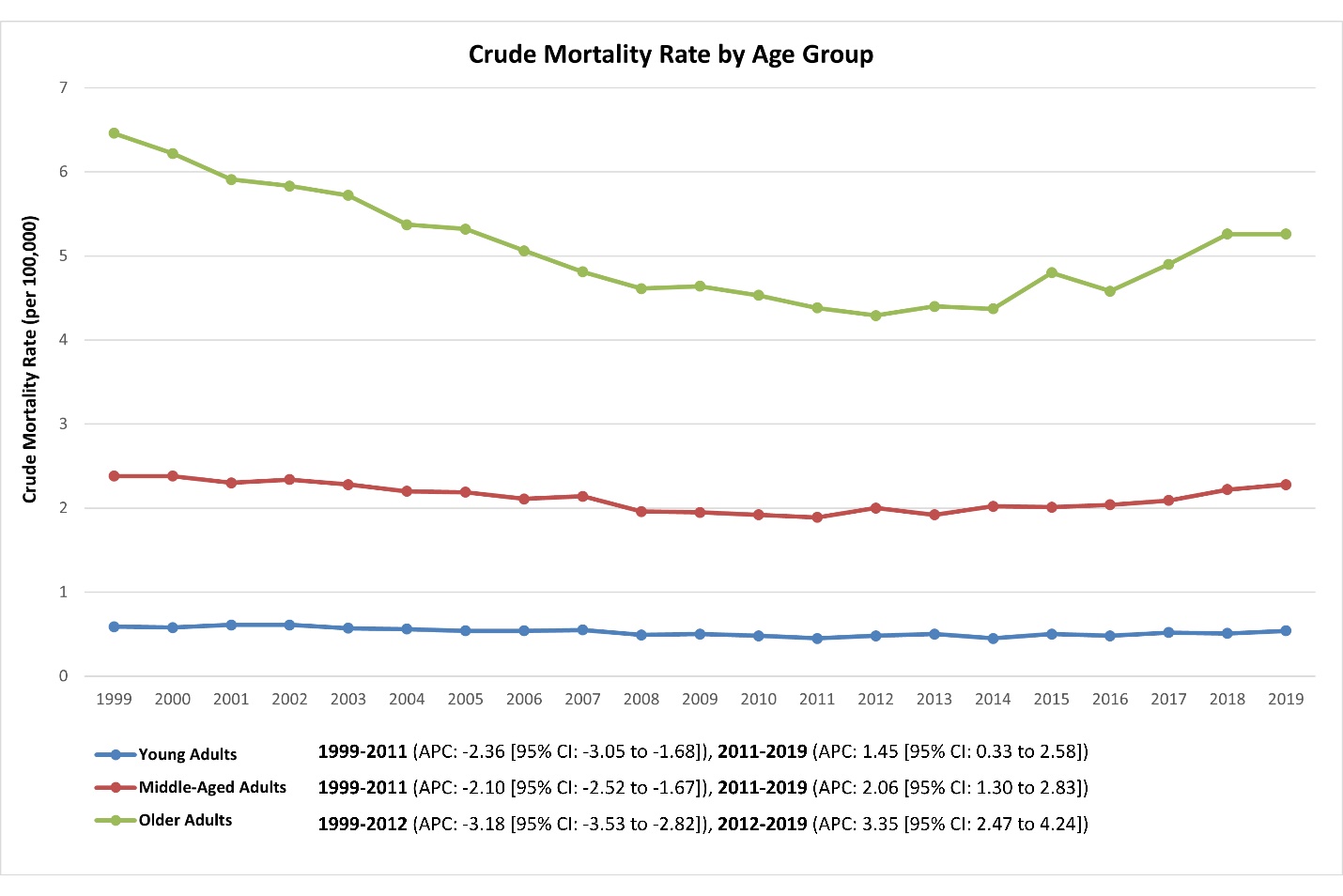


**Supplemental Figure 3.** Trends in PD-related mortality stratified by census region among adults in the United States from 1999 to 2019.


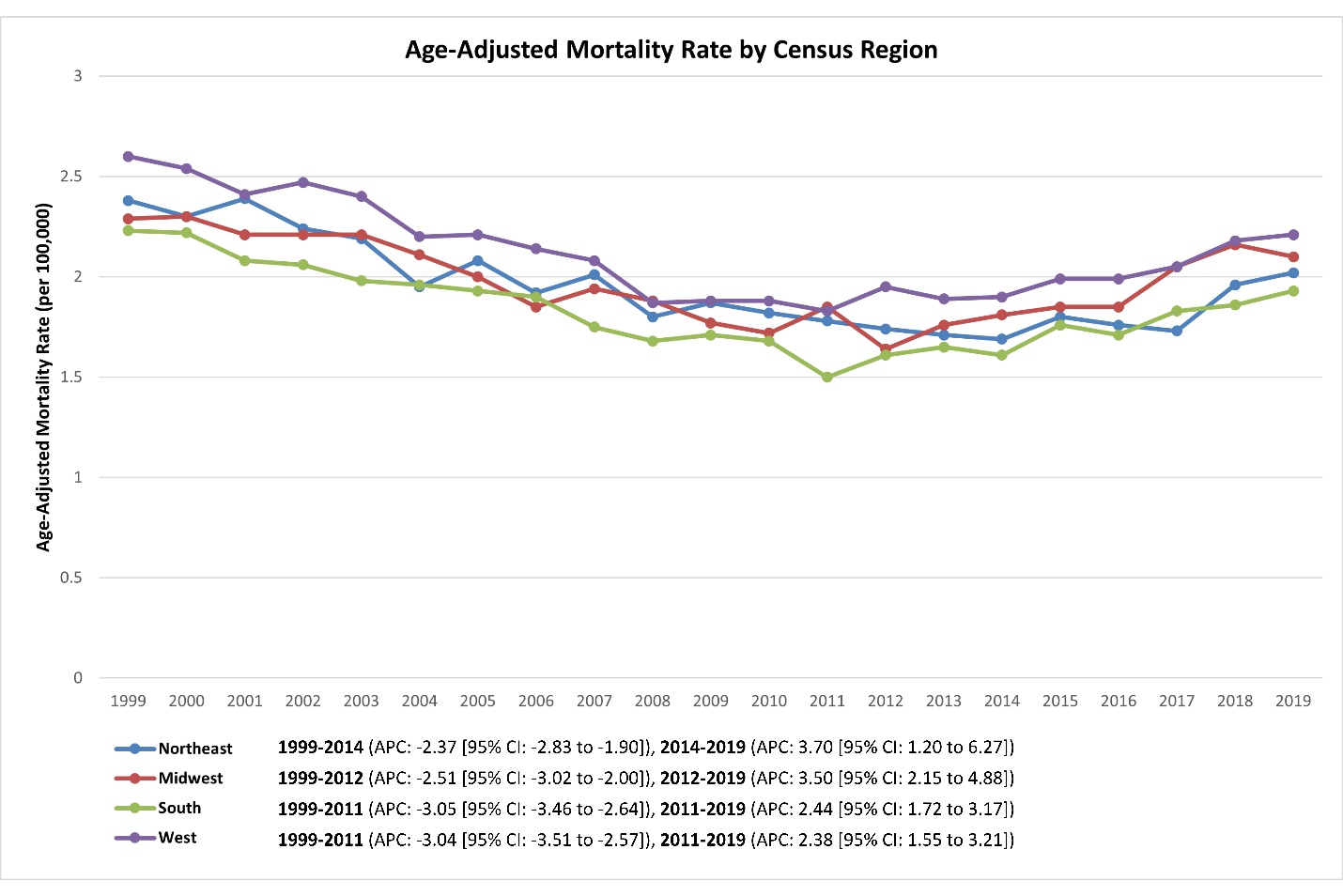

Supplement: Supplementary data [file mmc1.docx]
